# Microbial communities play important roles in modulating paddy soil fertility

**DOI:** 10.1038/srep20326

**Published:** 2016-02-04

**Authors:** Xuesong Luo, Xiaoqian Fu, Yun Yang, Peng Cai, Shaobing Peng, Wenli Chen, Qiaoyun Huang

**Affiliations:** 1 State Key Laboratory of Agricultural Microbiology, Huazhong Agricultural University, Wuhan 430070, China; 2 Key Laboratory of Arable Land Conservation (Middle and Lower Reaches of Yangtze River), Ministry of Agriculture, College of Resources and Environment, Huazhong Agricultural University, Wuhan 430070, China; 3 Crop Physiology and Production Center (CPPC), College of Plant Science and Technology, Huazhong Agricultural University, Wuhan Hubei 430070, China

## Abstract

We studied microbial communities in two paddy soils, which did not receive nitrogen fertilization and were distinguished by the soil properties. The two microbial communities differed in the relative abundance of gram-negative bacteria and total microbial biomass. Variability in microbial communities between the two fields was related to the levels of phosphorus and soil moisture. Redundancy analysis for individual soils showed that the bacterial community dynamics in the high-yield soil were significantly correlated with total carbon, moisture, available potassium, and pH, and those in the low-yield cores were shaped by pH, and nitrogen factors. Biolog Eco-plate data showed a more active microbial community in the high yield soil. The variations of enzymatic activities in the two soils were significantly explained by total nitrogen, total potassium, and moisture. The enzymatic variability in the low-yield soil was significantly explained by potassium, available nitrogen, pH, and total carbon, and that in the high-yield soil was partially explained by potassium and moisture. We found the relative abundances of Gram-negative bacteria and *Actinomycetes* partially explained the spatial and temporal variations of soil enzymatic activities, respectively. The high-yield soil microbes are probably more active to modulate soil fertility for rice production.

Rice is the major human food in Asia. Organic and chemical fertilizers are always applied in rice cultivation to increase rice yield. Soil microorganisms are crucial for nutrient cycling, soil fertility, and crop productivity^1^,^2^,^3^,^4^. Maintaining the health of soil microbiota is important for soil fertility and optimal crop yield.

Microbial communities within rice fields in different habitats vary in diversity and response to environmental changes. Microbial communities in the rice rhizosphere can be significantly affected by seasonal changes^5^. Fertilization treatments also affect soil properties and the microbial community in rice fields^6^. Chemical fertilizers are likely to stimulate the growth of gram-positive bacteria in rice soils, while organic amendments increase the relative abundance of bacteria and fungi and decrease the abundance of actinomycetes^7^,^8^. Nitrogen fertilizers increase soil biomass in organic carbon-rich rice soils^9^. Balanced fertilization increases microbial functional diversity in phosphorus-limited rice soils^10^. Overuse of urea in paddy fields may cause a decrease in microbial diversity^11^. Organic matter content, total nitrogen (TN), total phosphorus (TP), and even total potassium (TK) and/or available potassium (AK) restricted the activity, density, and structure of microbial communities in several paddy soils under different fertilization treatments in different habitats^7^,^12^,^13^,^14^,^15^. Organic fertilizers and reduced amounts of chemical fertilizers, i.e. a balanced fertilization program, were recommended for optimal microbial community development and improved rice soil quality^16^,^17^.

Nitrogen is generally the most limiting nutrient for rice production^18^. The application of inorganic ammonium-based nitrogen fertilizers has substantially increased rice yields but plant assimilation efficiency of nitrogen has shown a decreasing trend^19^. More than 50% of the applied nitrogen dissipates into the environment by volatilization, leaching, surface runoff, and denitrification^20^. This results in the pollution of fresh water and marine ecosystems^21^. Emissions of toxic ammonia^22^ into the atmosphere can cause health hazards such as gastric cancer and other diseases^23^. Reducing the amount of nitrogen fertilizer used is therefore an important measure that can improve the global nitrogen balance^24^. However, the dynamics of microbial communities in rice soils that do not use nitrogen fertilizer input and microorganism effects on soil fertility and rice productivity are not understood.

In this study, we evaluated the biomass, functionality, and structure of the microbial community in two nearby rice fields with different production using a multidisciplinary approach. The two fields used nitrogen-free fertilizer treatment for 7 years. The biomass and microbial community structure was obtained by analysis of phospholipid fatty acids (PLFA). Microbial metabolic potential in the soils was evaluated on the basis of the substrate utilization by Biolog Eco-Plate profiles. We found that the soil microbial community structure and function in the nitrogen-free fertilizer fields varied in its relationship to soil properties. An active microbial community in high-yield soil would be more beneficial for maintaining soil fertility and increasing rice yields.

## Results

### Soil parameters

Nitrogen-free fertilizers were used for seven years in soils from two nearby fields as paired comparisons in a rice productivity study. Each field possessed an area of 123.5 m^2^ (13 m × 9.5 m). The distance between the two fields was only approximately 30 m. The thirty-day-old seedlings of the same rice cultivar (Tianyou huazhan) were transplanted in the two soils (15 cm of the seedling stands in water). Plant spacing is 16.7 cm × 26.7 cm. The two fields received 80.0 kg ha^−1^ superphosphate and potassium chloride 150.0 kg ha^−1^ each year. The annual productivity of the two fields significantly differed by 1t ha^−1^ in seven years. The soil chemical properties significantly differed between the two soils (Table 1). The concentrations of TN (F = 256.72, P < 0.05), TP (F = 35.75, P < 0.05), total carbon (TC) (F = 144.43, P < 0.05), available phosphorus (AP) (F = 21.64, P < 0.05) and AK (F = 61.00, P < 0.05) were significantly higher in samples of the high-yield field (HF) than those of the low-yield field (LF) during rice growth periods. Soil pH ranged from 4.92 to 5.68. Sample moisture (MOI) differed in the two fields during the panicle differentiation and full-heading periods. TK content and C/N ratio of samples only differed during the panicle differentiation stage. These data suggested higher soil fertility in the HF. The two soils had identical climate exposure, and have the same soil type and crop (rice) so the differences between the two soils could be related to the initial microbial community and historical soil properties.

### PLFA profiles characterizing soil microbial communities

The amounts of total PLFAs, an indicator of microbial biomass, and PFLAs from Gram-negative bacteria (GN), were significantly higher in the HF soils (Table 2). The abundances of Gram-positive bacteria (GP) and fungi PLFAs showed the opposite trend. The percentages of anaerobic bacteria and AM fungi PLFAs were significantly different in different rice growth stages. Abundances of GP, GN, and *Actionomycetes* PLFAs were more sensitive to rice growth stages in the HF than those in the LF. However, eukaryote PLFAs abundances in both soils were relatively stable. These data indicated that the two soils had different microbial communities with different responses to the same environmental variables. Significant variations of the microbial community in the rhizosphere and bulk soils occurred during the rice growing season. This variation rapidly disappeared after harvest, suggesting that the variation was related to the rhizosphere recruiting effect from the growing rice. ANOVA analysis showed that the soil microbial communities were significantly affected by the geographic site, rice growing stage, or their interaction (Table 2).

**Figure 1:**
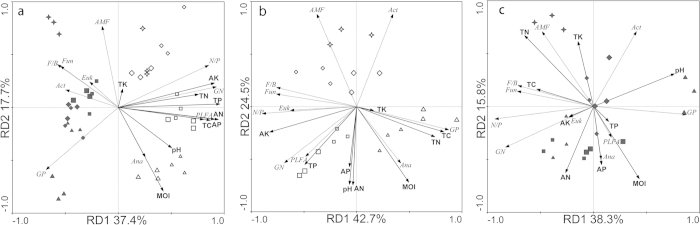
Redundancy analysis (RDA) for PLFA composition along soil fertility gradients (the first two components are shown) for the two soils (**a**), at only the high yield soil (**b**), and at the low yield soil (**c**). Four symbol types were used: square for panicle differentiation stage, triangle for full-heading stage, diamond for maturity stage, and a star for after-harvest. Solid and hollow represented samples from high yield and low yield fields, respectively. GP, gram-positive bacteria; GN, gram-negative bacteria; Fun, fungi; AMF, AM fungi; Act, actinomycetes; Ana, anaerobe; Euk, eukaryote.

The redundancy analysis (RDA) result illustrated the relationship between the environmental variables and the soil PLFA contents. As shown in Fig. 1a, we found the microbial communities in the two soils were clearly separated. The microbial communities in the HF soil were significantly different in the different rice growing stages, suggesting a close link between the rice stage and microbial community dynamics. The first and second axes explained 37.4% and 17.7% of the total PFLA variability, respectively. The RDA ordination plot shows that microbial communities from the same fields were clustered together. Axis 1 separated microbial communities of the HF from those of the LF, and samples from different periods were separated by axis 2. Significant difference (p < 0.05) was observed among different periods in the two fields when per mutational tests were analyzed based on PLFA contents. Increases in total PLFA and the percentage of GN and anaerobic bacteria PLFAs in HF along axis 1 were positively correlated with all soil physicochemical factors. However, increases in the percentage of GP, fungal, and actinomycete PLFAs in field L along axis 1 were negatively correlated with all soil physicochemical factors. This indicated that the soil physicochemical factors are associated with the soil microbial community and are significantly affected by TP (explained 35% of the total variation) and MOI (explained 14% of the total variation) (Table 3).

Interactions between soil environmental variables and soil microbial community composition within individual soils were evaluated. Distinct microbial community clusters were related to the rice growing periods. This suggested the microbial communities in both fields were both highly modulated by temporal factors. In the HF, the RDA explained 76% of the variation in the PLFA data of which 67.2% was explained by the first two axes (Fig. 1b). Increases in the percent of GP PLFAs along axis 1 (42.7% of the total variation) were positively correlated with TC and TN. Increases in total PLFA, the percent of GN and anaerobic bacteria PLFAs in the HF along axis 2 (24.5% of the total variation) were positively correlated with AP, TP and MOI. Clusters of samples reflected the rice growth periods. The significant factors affecting the microbial community composition were TC (explaining 31% of the variation), MOI (explaining 16% of the variation), AK (11%), and pH (8%) (Table 3). In the LF, the model accounted for 68.4% of the total variation (Fig. 1c). Increases in the percent of GP PLFAs along axis 1 (38.3% of the total variation) were positively correlated with pH, but were negatively correlated with TC and TN. In contrast, increases in the percent of GN PLFAs along axis 1 were negatively correlated with pH. Increases in the total PLFA and the percent of anaerobic bacteria PLFAs along axis 2 (15.8% of the total variation) were positively correlated with C/N and moisture (MOI). The largest percentage of variation was explained by pH (25%), influencing variation on both axes. The nitrogen factors (TN plus AN) explained 28% of the total variation (Table 3). RDA plots of two fields both indicated that AM fungal, fungal, and actinomycete PLFAs along axis 2 were negatively correlated with MOI and available nutrients. These data clearly indicate differences in the microbial community modulating factors between soils from the two fields. In the HF, the dynamic supply of C and MOI significantly modulated shifts in the microbial community, while in the LF, nitrogen loss and pH variation influenced microbial community dynamics. A closer linkage between the microbial community and soil fertility factors was seen in the HF.

### Community-level physiological profiles (CLPPs)

**Figure 2:**
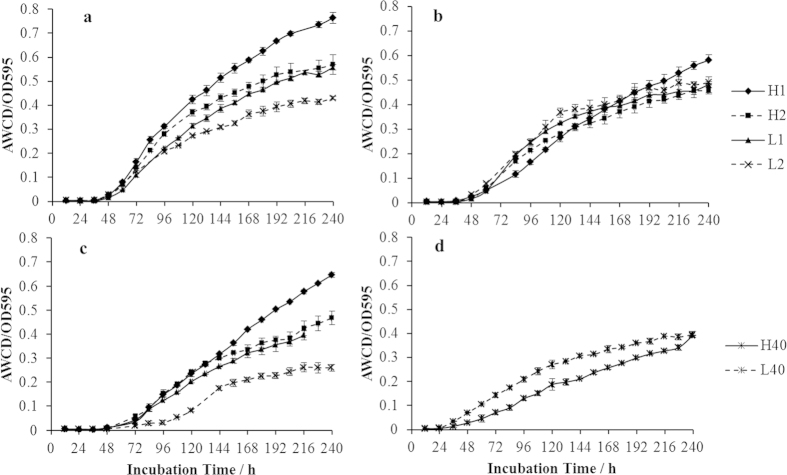
Average well color development (AWCD) for soil microbial communities in panicle differentiation stage (**a**), Full-heading stage (**b**), Maturity stage (**c**), and Postharvest (**d**). 1, Rhizosphere samples; 2. Non-rhizosphere samples; 0, Homogeneous samples. H, high-yield soil microbial community; L, microbes from low-yield soil.

Average well color development (AWCD) indicated the microbial activity from fast growing heterotrophs (mainly composed of rapid growing gram-negative bacteria) (Fig. 2). The HF soil had a faster rate of AWCD increase compared to the LF, especially during the panicle differentiation and maturity stages (Fig. 2a,c). In the full-heading stage, soil microbial communities in all samples had similar activity (Fig. 2b). After harvest, the activity in both soils decreased (Fig. 2d). The microbial activity in the rhizosphere was greater than the bulk soil in the panicle differentiation and maturity stages. This suggests that rice roots stimulated the growth of fast growing heterotrophs.

PCA of the Biolog Eco-plate data indicated distinct quantitative and qualitative differences of the fast growing heterotrophs between the two fields in use of 31 carbon sources (Table 4). In the HF, the first two principal components only accounted for 21.3% and 14.1% of the total variations, respectively. Single carbon source substrate utilization (SU) patterns of samples from the panicle differentiation stage were significantly different from samples after harvest. Differentiation along the first principal component (PC1) was primarily due to polymerases and carbohydrates. Differentiation along the second principal component (PC2) was due to the utilization of amines and carboxylic acids. In the LF, the PC1 and PC2 accounted for 22.2% and 20.9%, respectively. SU patterns of bulk soil in the full-heading period were significantly different from those of samples in other growth stages. Variability in the PC1 was explained by the utilization of phenolic acids and carbohydrates, while differentiation along the PC2 was primarily due to amino acids and carbohydrates.

We calculated indexes for microbial function diversity (Table 5). Shannon indexes showed that functional diversity of the fast growing heterotrophs in the HF was slightly higher than that in the LF, and McIntosh indexes showed that such functional diversity of microbes was more uniformly distributed in HF. The diversity, richness, and evenness of the functionality indicated by the microplate data were affected by the interaction between sample site and growth stage (P < 0.01) (Table 5). This indicated the function of the fast growing heterotrophs in the two soils differed by their carbon source utilization potentials and their carbon sources utilization potential would be greatly influenced by temporal factors including weather changes and factors associated with rice production.

### Soil enzymatic activities

Soil enzymatic activities were also indicators of soil microbial function. Acid phosphatase activity in the HF soils was lower than in the LF (Table 6). Invertase activities for both soils were similar during most of the rice growing periods. Urease activities were highly variable during the rice growth stages. Arylsulfatase activities were similar between the two soils, but the activity was higher in the LF after harvest, suggesting differences in function of the original microbial community. ANOVA analysis indicated that acid phosphatase, urease, and invertase activities were affected by the interaction effects of field difference and rice growth stage (p < 0.05), while aryl sulfatase activities were significantly affected by the field (p < 0.05) and growth stage (P < 0.05) independently. These results indicated that although the microbial community in the two soils was different, the plant recruiting microbial function in the soils was similar.

**Figure 3:**
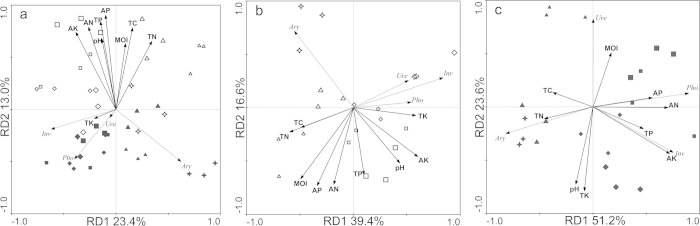
Redundancy analysis (RDA) of the enzyme activity data on soil properties at the two soils (**a**), high-yield (H) (**b**) and low-yield (L) soils (**c**). Square for panicle differentiation stage, triangle for full-heading stage, diamond for maturity stage, star for after-harvest. Solids and hollows represent samples from H and L soils, respectively. Ure, Urease activity; Pho, Alkli phosphatase activity; Ary, Aryl sulfatase; Inv, Invertase.

When soil physicochemical factors were used to constrain the ordination of all four enzymes with RDA (Fig. 3a), the model accounted for 47.8% of the total variation. Axis 1 (23.4% of the total variation) largely differentiated aryl sulfatase vs. urease, acid phosphatase, and invertase. Increases in all enzyme activities along axis 2 (13% of the total variation) were negatively correlated with all soil physicochemical factors. Soil samples of field H were separated from those of field L along with the axis 2. The main driving factors behind variation in microbial community composition were soil chemical factors including TN (15%), influencing the variation on both axes, followed by AK and MOI, which together explained 18% of the variation (Table 3). These results indicate that soil property variables were partly linked to soil enzymatic activities.

Redundancy analysis (RDA) was performed to explore interactions between soil temporal variables and enzyme activities. In the HF, the model accounted for 65.5% of the total variation (Fig. 3b). Axis 1 (39.4% of the total variation) largely differentiated aryl sulfatase vs. urease, alkali phosphatase and invertase. Increases in all enzyme activities along axis 2 (16.6% of the total variation) were negatively correlated with all soil physicochemical factors. Clusters of samples reflected the rice growth periods. The RDA plot suggested that enzyme activities were significantly positively correlated with (listed in order of decreasing importance) TK, MOI, and AK (Table 3). In the LF, the model accounted for 80.9% of the total variation (Fig. 3c). Axis 1 (51.2% of the total variation) largely differentiated aryl sulfatase vs. urease, acid phosphatase and invertase. Activities of urease and acid phosphatase loaded positively on axis 2 (23.6% of the total variation) and were associated with soil MOI and AP, respectively. Activities of aryl sulfatase and invertase loaded negatively on axis 2 and were associated with soil TN and AK, respectively. Clusters of samples reflected the rice growth periods. Such variation was explained by AK (32%), followed by TK (16%), AN (15%), and pH (7%) (Table 3). These data suggest that in the LF soil both K and N restricted the enzymatic activities produced by organisms.

### Correlation with enzyme activities and microbial communities

To further study the link between soil enzymatic activities and residential microbes, we analyzed the relationship between soil enzymes and the microbial community. Initially, the enzymatic activities were divided by the total PLFA contents. This will normalize the biomass of each microbial community. And then, we can determine linkage between community structure and enzymatic activities^25^.

**Figure 4:**
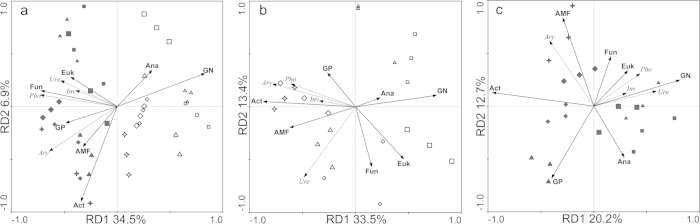
Redundancy analysis (RDA) of the enzyme activity data on soil microbial community data at the two soils (**a**), high-yield soil (**b**), and low-yield soil (**c**). Four symbol types were used: square for panicle differentiation stage, triangle for full-heading stage, diamond for maturity stage, star for after-harvest. Solid and hollow represented samples from H and L soils, respectively. Ure, Urease activity; Pho, Alkli phosphatase activity; Ary, Aryl sulfatase; Inv, Invertase.GP, gram-positive bacteria; GN, gram-negative bacteria; Fun, fungi; AMF, AM fungi; Act, actinomycetes; Ana, anaerobe; Euk, eukaryote.

When the PLFA data were used to constrain the ordination of treated data of enzymatic activities with RDA (Fig. 4a), the model accounted for 43.2% of the total variation. The first ordination RDA axis explained 34.5% of the total variability of the PLFA, while the second ordination RDA axis explained 6.9%. The RDA ordination plot shows that blots representing the enzymatic activities from the same field were clustered together. Axis 1 separated spots from the HF from those of the LF and spots representing microbial community from different periods were separated by axis 2. Increases in all enzyme activities along axis 1 were negatively correlated with the percentage of GN and anaerobic bacteria PLFAs. Increases in urease, acid phosphatase, and invertase along axis 1 were positively correlated with the percentage of fungal PLFAs. Increases in aryl sulfatase along axis 1 were positively correlated with the percent of actinomycetes PLFAs. The PLFA contents changed temporally. The main sources of enzymatic activity variation were GN (explaining 25% of the total variation) and fungal (explaining 8% of the total variation) (Table 3). This indicated the microbial community influenced the enzymatic activities in the two soils.

Individual RDA for each soil showed that in the HF, 54.7% of the variation was explained. A total of 46.9% of this variability was explained by the first two axes (Fig. 4b). Increases in all the enzyme activities along axis 1 (33.5% of the total variation) were positively correlated with the percentage of actinomycetes PLFAs, which explained 26% of the total variation (Table 3). In the HF soil, acinomycetes dynamics may play important roles in modulating soil enzymatic activities, while the enzymes produced by other microbes could be redundant. In the LF soil, the model only accounted for 38.7% of the total variation (Fig. 4c). *Actionomycetes* PLFA also explained 19% of the total variations (Table 3). These results indicated variability in *Actionomycetes* activity contributed to modulating the temporal shift of the enzymatic activities in both soils. A closer linkage between soil enzymatic activities and microbes occurs in the HF because the total variation explained was greater.

## Discussions

A lot of previous studies indicated soil fertility factors affected microbial communities^26^,^27^,^28^,^29^,^30^,^31^. How soil microbes modulate soil fertility remains an opening question. A reduction in the use of nitrogen fertilizers may help improve the global N balance. However, nitrogen free-fertilization combined with continuous cropping can lead to nitrogen depletion and produce deleterious effects on the crop ecosystem. The relationship between the microbial community and soil fertility in the nitrogen free fertilizer management soils was unclear.

We focused on the response of microbial communities to rice growing stages in geographically close related rice soils that didn’t receive nitrogen fertilizers. The two neighboring soils, which possessed the same soil type, the crop (rice), and the agriculture use history, were employed as duplicates for a long-term “nitrogen-free fertilization” experiment, and the annual productivity significantly differed during the experiment. Soil properties data demonstrated that the indicators of soil fertility in the HF soil were higher than those of the LF soil. Pedologists were surprising to see this phenomenon.

PLFA analysis, Biolog Eco-Plate assays, and enzymatic activity measurements, are often used to investigate the general structure and the functional potential of soil microbial communities^32^,^33^,^34^,^35^,^36^. PLFA analysis is a powerful tool used to study microbial community response to environmental variables. Our PLFA profiles showed that microbial communities differed in the two soils. The total PLFAs, an indicator of microbial biomass, and the PLFA possessed by GN were more abundant in the HF soils in contrast to GP bacteria and fungi. Rice growth stages significantly affected the percent of anaerobic bacteria and AM fungi. Bacteria were more sensitive to plant growth stages in the high-yield field; while eukaryotes in both soils were relatively stable in the relative abundance (Table 2).

RDA analysis of the samples from our fields showed that relative abundance of GN PLFAs was correlated with the TP, TC, TN, AP, and AK. The content of PLFAs from GP, AM fungi, and actinomycetes was negatively correlated with soil chemical properties. Fungi tend to inhabit drier soils^37^,^38^. It is therefore not surprising that fungus PLFAs were negatively correlated with moisture content (Fig. 1a). The percentage of anaerobic bacteria PLFAs was positively correlated with MOI, TP, AK and pH. TP and MOI were the main factors shaping the PLFA profiles in soil samples (p < 0.001). AK and pH also influenced the microbial community but to a lesser degree (p < 0.05). The microbial communities in the two soils were distinctive and were largely determined by soil properties. Despite microbial community differences between sites, the total PLFA was correlated with phosphorus variables.

Interactions between soil environmental variables and soil microbial community composition within individual soils were evaluated by RDA (Fig. 1b,c). Microbial community clusters were found to be related to rice growing periods. This suggests the microbial communities in both fields were highly influenced by temporal change. In the HF, the dynamic supply of C, MOI, AK, and pH significantly influenced changes in the microbial community. In the LF, N variables had the greatest effects on microbial community differentiation. The microbial community in the more HF soil is relatively more active in modulating soil fertility because there is a more significant relationship between soil properties and the microbial community.

The Biolog Eco-plate data provided potential carbon utilization activities for fast growing heterotrophic bacteria. A relatively higher respiratory activity was observed from the HF soils, especially during the panicle differentiation and maturity stages (Fig. 2a,c). PCA of the Biolog Eco-plate data distinguished the rapid growing heterotrophic microbes in the two fields by variability in their carbon sources used (Table 4). Both geographical location and the plant growth stage significantly affected the carbon source utilization profile of the rapid growing heterotrophic microbial community. This was indicated by the significant variations in the diversity indexes calculated from the Biolog data. The potential functions of these microbes are therefore determined by both the geographic sites and the plant growing stages. These data support our hypothesis that the two soils possessed different microbial communities. The rapid growing heterotrophs in HF soil are more active and functionally redundant.

We measured the activities of urease, acid phosphatase, invertase, and aryl sulfatase. These enzymes drive N, P, C, and S cycles in soils, respectively^39^,^40^. Previous findings suggested that soil enzymatic activities are associated with carbon factor, site specific soil moisture and nitrogen fertilizers^41^,^42^. In this study, we found the activities of urease and acid phosphatase in the high-yield and low-yield soils were significantly different although both fields received the same fertilizers (without nitrogen) (Table 6). The invertase activity was negatively correlated with TC and TN; aryl sulfatase activity was negatively correlated with AN and TK; urease activity was negatively correlated with TN; acid phosphatase activity was negatively correlated with TC, TN, TP and AP (data not shown). Although a negative relationship between enzyme activities and N, P availability has been found in other soils^43^,^44^,^45^, we found that potassium is also an important factor in modulating soil enzymatic activities. In the HF soil, we found a higher nutrient levels and a lower enzymatic activity. This suggested the nutrient supply for microbes would be more redundant.

RDA of soil environmental variables and enzyme activities indicated that K variables could be the key factors modulating soil enzyme activities in the HF. N limitation likely restricted the enzymatic activities of organisms in the LF (Fig. 4a and Table 3). We also found the soil properties less explained the enzymatic variation from the HF soils. Considering the nutrient level, microbial biomass (indicated by the total PLFA content) and rapid-growing heterotrophic microbial activity in the HF soil was higher, we hypothesize that the microbes in HF soils would be more active to modulate soil enzymatic activities rather than be passively controlled by nutrient levels. To test this hypothesis, RDA analysis of the relatedness between soil enzyme activities and the microbial community suggested that the microbe community may help regulate soil enzymatic activity. We found a negative correlation between enzyme activities and the percentage of GN, *Actinobacteria* and fungal PLFAs. These factors explained most of the total variation (Fig. 4a). Individual RDA for each soil showed that the relative abundance of actinomycetes PLFAs was the most important factor explaining the temporal variation. The correlations between enzyme activities and microbial community differed in the two soils (Fig. 4b,c). Increases in acid phosphatase and invertase activities were negatively correlated with the percentage of GN and anaerobic bacteria, fungi and eukaryotes PLFAs in the HF. This correlation showed an opposite trend in the LF. These results indicate that enzymatic activities in soils can be differently modulated by temporal shifts in the microbial community. The relatively greater enzymatic variation explained by microbial community data in the HF soil demonstrated a more active association between enzymatic activities and microbes.

In summary, we found that both soil factors and rice plant growth stages contributed to the temporal and spatial structure of the functional microbial communities in the two rice soils. Variation in the microbial community is correlated with the enzymatic activities and soil fertility dynamics. This indicates that microbes play important roles in determining the fertility of nitrogen-free fertilizer rice soils. The microbial community in high-yield soil was more responsive to changes in soil properties, more effective at modulating soil enzymatic activities that were less associated with soil nutrient levels.

Although the PLFA analysis, Biolog Eco-Plate assays, and enzymatic activity measurements are traditionally used to investigate the general structure and the functional potential of soil microbial communities, these methodologies are not able to provide either the taxonomic information from the microbial community or probe the function from specific microbial group. Further analysis of the microbial community and function using molecular biology approaches will help us better understand the mechanisms by which the microbial community modulates soil fertility.

## Materials and Methods

### Soil sampling

Soil samples were collected in 2013 from two rice fields, receiving no nitrogen fertilizer, located in Zhougan Village (115°33′E, 29°51′N), Dajin Town, Wuxue City, Hubei Province, China. This area has a subtropical monsoon climate with an annual rainfall of 1360 mm and annual mean temperature of 17.6 °C. The two soils are sandy clay loams with 22.29% silt, 20.68% clay, and 57.03% sand, according to the International System of Soil Texture Classification Standard. The zero nitrogen fertilizing double-harvest rice system was experimentally introduced (40.0 kg ha^−1^ superphosphate and potassium chloride 75.0 kg ha^−1^). The annual rice yield between the two soils differed by 1 t ha^−1^.Thus, the high-yield field was referred to as the HF, and the low yield one was the LF.

In this study, soil samples (0–15 cm) were collected on August 24 (Panicle differentiation stage), September 14 (Full-heading stage), October 5 (Maturity stage) and November 5 (Post harvest). Rhizosphere and non-rhizosphere samples were collected. A total of 9 random soil cores within each plot (30 m^2^) were mixed to provide one sample^46^. Three replicate samples were collected for each representative time-site point. Each sample was partitioned into three subsamples: one was stored at −80 °C for PLFA extractions, one was partially air-dried and passed through a 2 mm sieve for chemical analysis, and one was stored at 4 °C for up to 7 d prior to analysis of biological characteristics.

### Soil parameters

Air-dried samples were sieved to < 2 mm and used to determine soil texture, TC, TN, TP, TK, AN, AP, AK concents, MOI, and pH. Measurements of soil characteristics were conducted using the methods of Lu^47^. Soil moisture content was measured by calculating the weight of lost water after the sample was dried at 105 °C for 24 h. Soil pH was determined in a soil suspension possessing a soil: water ratio of 1: 2.5 (w/v) with a pH meter (UB-7,UltraBASIC, Denver, CO). Soil TC and TN concentrations were measured by dry combustion analysis using a Vario MAX-CN Elemental Analyzer (Elementar, Germany). AN was measured by the alkali-hydrolysis and diffusion method^48^. TP and TK was extracted and determined by the perchloric acid digestion methods and spectrophotometry protocols^49^,^50^. AP was determined following the methods described by Olsen *et al.*^51^. AK was extracted with 1 M NH_4_OAc (1:10 soil: solution ratio) for 30 min and analyzed using atomic absorption spectrophotometry^52^.

### Soil enzyme activities

Four types of soil enzymes (invertase, urease, acid phosphatase, and aryl sulfatase) were selected as indicators of microbial capacity to drive nutrient cycling (C, N, P, and S, respectively).

Invertase activity was determined by assessing the reducing sugars released after samples were incubated with 8% saccharose in phosphate buffer (pH 5.5) at 37 °C for 24 h^53^. Invertase activity was measured at a wavelength of 508 nm using a spectrophotometer (Beijing Dongxun the Heavens and the Earth Instrument Co.Ltd).

Urease activity was estimated following the method of Hoffmann^54^, using citrate phosphate buffer (pH 6.7) and 10% urea solution ratio of 1:4:2 (w/v/v) as a substrate. The mixture was incubated at 38 °C for 3 h and activity was measured at a wavelength of 578 nm using a spectrophotometer.

Acid phosphatase activity was detected as follows: A 2.5 g dried soil sample was incubated with 5 ml 0.5% disodium phenyl phosphate and boric acid buffer (pH 5.0) at 37 °C for 12 h. The p-nitrophenol released during enzymatic hydrolysis was determined using a spectrophotometer at a wavelength of 570 nm^55^.

Aryl sulfatase was essayed by measuring the released nitrophenol by nitrophenol potassium sulfate. A 0.5g soil suspend in a mixture with 0.005 M nitrophenol potassium sulfate and 0.5 M acetic acid buffer ratio of 1:1:4 (w/v/v) and incubated at 37 °C for 1 h. The activity was measured at 400 nm by a spectrophotometer.

### Community-level physiological profiles (CLPPs)

The capability of soil microbial communities to utilize a variety of individual carbon sources was assessed using BIOLOG-ECO plates (Biolog, Inc., USA). Each ECO plate contained three replicate wells of 31 different carbon sources, including carbohydrates, carboxylic acids, amino acids, amines, polymers, phenolic acids, and a control. The rate of utilization of the carton sources is linked to the production of NADH, which reduces tetrazoliun, a redox indicator dye that changes from colorless to purple. Soil samples were suspended in (1:9 ratio) sterile saline solution (0.85% w/v NaCl) on votex for 30 min. Then 1 mL of soil suspension was transferred into a microcentrifuge tube and centrifuged at 10,000 rpm for 20 min. The supernatant was removed. The pellets were washed twice to remove water soluble carbon using the sterile saline solution and resuspended in 20 mL of the same solution. A 150 μl sample of the suspension was inoculated into each well. The plates were incubated at 20 °C and read calorimetrically according to the protocols described by Garland *et al.*^25^. Color development in each well was recorded as optical density (OD) at 595 nm and 750 nm with an ELISA plate reader at 12 h intervals for 240 h.

The well absorbance values were adjusted by subtracting the absorbance of the control well (water only) before the data analysis; negative readings (OD < 0) were excluded from all subsequent analysis. Microbial activity in each microplate, expressed as average well color development (AWCD) was determined as follows: AWCD = ∑OD_i_/31, where OD_i_ is the optical density value from each well. The 180 h OD value for each sample in triplicate, divided by their AWCD to normalize the values were used to calculate the functional diversities using Shannon index and Shannon evenness^56^,^57^. The Shannon index is calculated as follows: H = −∑Pi(ln pi), where pi is the ratio of the activity on each substrate (OD_i_) to the sum of activities on all substrates (∑OD_i_). The evenness was calculated as E = H/ln (richness), where richness referred to the number of substrates utilized.

### PLFA analysis

A 2 g quantity of freeze dried soil was extracted with 15.8 mL single-phase chloroform-methanol-aqueous buffer system (Chloroform: methanol: citric acid = 1:2:0.8)^58^. Phospholipids were collected in the methyl esters by mild alkaline methanolysis to form fatty acid methylesters (FAMEs)^59^. The FAMEs were identified and quantified using an Agilent 6850 Series capillary gas chromatograph (Agilent Technologies, Wilmington, DE). Identification and quantification of FAMEs was conducted using MIDI software with MIDI microbial calibration standards (MIDI, Inc., Newark, DE). Absolute amounts of FAMEs (nmolg^−1^) were calculated using 19:0 internal standards^60^ and these values were subsequently used to calculate the relative abundance of individual components. Individual PLFA were used to indicate broad groups of the microbial community: 18:1ω9c, 18:2ω6c, and 18:3ω6c for fungi; 16:1ω5c for AM fungi; 3OH15:0, 2OH16:0, and 2OH18:0 for anaerobic bacteria^61^; 16:0 10 methyl for actinomycetes^62^ and 20:4ω6c, 20:4ω9c for eukaryotes. The fatty acids i15:0, a15:0, i17:0, and a17:0 were taken to represent the GP^60^, and the fatty acids cy17:0, cy19:0, 18:1ω9t represented the GN. The bacterial sums were calculated using both GP and GN markers^27^.

### Statistical analyses

The statistical analyses of data were conducted in the SPSS software program (ver. 17.0 for Windows, Chicago, IL, USA). Variation among samples for different treatments and rice growth stages were analyzed using ANOVA. Levene’s test was used to assess the equality of variances before performing ANOVA, and significant differences between the treatments and growth stages were determined by the SNK test. The differences were considered statistically significant when P < 0.05. PCA was used to analyze the substrate utilization pattern based on the Eco-plate data. The diversity indexes variation were also analyzed by ANOVA. PLFA profiles and enzyme activities were compared using redundancy discriminate analysis (RDA) with Monte Carlo permutation test (CANOCO, for windows version 4.5, Microcomputer Power, Ithaca, USA). The Monte Carlo tests were based on 9999 random permutations of the data to explore significance of the environmental variables^63^. Soil chemical factors potentially affecting community structure and enzyme activities were used as the restricted variables. Microbial community data potentially affecting the normalized enzymatic activities were also used as the restricted variables where necessary.

## Additional Information

**How to cite this article**: Luo, X. *et al.* Microbial communities play important roles in modulating paddy soil fertility. *Sci. Rep.* **6**, 20326; doi: 10.1038/srep20326 (2016).

## Figures and Tables

**Table 1:** Chemical properties of the rice paddy soil samples.

Stage	Sample Type	TP (mg/kg)	TK (mg/kg)	AP (mg/kg)	AK (mg/kg)	AN (mg/kg)	MOI (%)	TN (%)	TC (%)	C/N
Panicle differentiation	H11	1118 ± 24 b	2275 ± 197 a	29.87 ± 1.29 b	183.91 ± 11.80 c	145.55 ± 6.99 c	36.02 ± 1.08 b	0.23 ± 0.00 c	2.32 ± 0.01 c	10.18 ± 0.02 c
	H12	1101 ± 69 b	2726 ± 56 b	29.60 ± 5.18 b	149.09 ± 19.00 b	150.88 ± 1.56 c	36.30 ± 0.67 b	0.23 ± 0.00 c	2.34 ± 0.00 c	10.27 ± 0.00 c
	L11	723 ± 104 a	2024 ± 50 a	14.68 ± 2.74 a	68.73 ± 6.17 a	110.64 ± 0.63 a	33.66 ± 0.69a	0.18 ± 0.00 a	1.71 ± 0.00 a	9.64 ± 0.04 a
	L12	736 ± 11 a	2359 ± 285 a	13.76 ± 2.93 a	65.84 ± 12.12 a	123.72 ± 4.37 b	32.48 ± 0.93 a	0.19 ± 0.00 b	1.84 ± 0.08 b	9.88 ± 0.16 b
Full-heading	H21	1004 ± 47 b	2531 ± 149 a	27.77 ± 1.56 b	110.06 ± 17.11 b	138.98 ± 10.01 b	33.60 ± 0.50 b	0.26 ± 0.00 c	2.73 ± 0.02 d	10.68 ± 0.11 a
	H22	1084 ± 36 c	2528 ± 170 a	31.93 ± 9.11 b	129.61 ± 22.71 b	145.33 ± 5.49 b	44.42 ± 1.48 c	0.25 ± .00 c	2.66 ± 0.01 c	10.55 ± 0.20 a
	L21	715 ± 23 a	2293 ± 362 a	11.98 ± 1.82 a	39.19 ± 4.74 a	106.86 ± 4.81 a	35.29 ± 0.72 a	0.17 ± 0.00 a	1.78 ± 0.00 a	10.39 ± 0.14 a
	L22	658 ± 20 a	2149 ± 74 a	10.97 ± 1.83 a	40.38 ± 6.25 a	111.04 ± 6.60 a	36.32 ± 0.44 a	0.19 ± 0.00 b	1.94 ± 0.01 b	10.17 ± 0.17 a
Maturity	H31	1053 ± 48 b	3010 ± 270 a	20.94 ± 3.46 b	151.45 ± 8.10 b	128.94 ± 2.41 b	31.07 ± 0.48 a	0.22 ± 0.00 b	2.38 ± 0.02 c	10.62 ± 0.27 a
	H32	1071 ± 14 b	2721 ± 182 a	28.99 ± 1.93 c	178.26 ± 22.06 b	137.53 ± 3.37 c	32.65 ± 2.48 a	0.23 ± 0.00 b	2.42 ± 0.02 c	10.38 ± 0.29 a
	L31	731 ± 12 a	2837 ± 217 a	12.98 ± 3.70 a	75.29 ± 16.28 a	112.76 ± 2.07 a	32.18 ± 0.40 a	0.18 ± 0.00 a	1.81 ± 0.00 a	9.84 ± 0.16 a
	L32	711 ± 22 a	2872 ± 181 a	11.73 ± 2.76 a	58.07 ± 13.78 a	117.58 ± 7.41 a	33.87 ± 0.50 a	0.18 ± 0.01 a	1.88 ± 0.02 b	10.34 ± 0.33 a
Background	H40	1011 ± 51	2027 ± 52	21.72 ± 4.50	120.44 ± 12.39	130.16 ± 6.72	29.29 ± 2.64	0.23 ± 0.01	2.31 ± 0.04	10.17 ± 0.30
	L40	674 ± 21	2669 ± 20	9.69 ± 1.95	51.24 ± 6.07	103.64 ± 2.80	26.97 ± 1.50	0.23 ± 0.00	1.86 ± 0.02	8.13 ± 0.23

Sample types in all tables were indicated as follows: H()1were rhizosphere soil from HF at indicated time points; H()2, bulk soil from HF; L()1, rhizosphere soil from LF, L()2, bulk soil from LF. X ( = 1, 2, 3 or 4) indicate the corresponding stages. L/H40 indicate soil samples collected after harvest.

The data are means ± SD (n = 3). Different letters with NSK tests indicate significant differences (p < 0.05).

**Table 2:** Soil microbial diversity evaluated using analysis of variance (ANOVA).

Stage	Sample Type	Total PLFA (nM/g)	GP (%)	GN (%)	Ana (%)	Act (%)	AMF (%)	Fun (%)	Euk (%)	Bac (%)
Panicle differentiation	H11	236.99 ± 1.25 b	28.26 ± 0.76 a	47.37 ± 0.45 d	1.12 ± 0.01 b	9.54 ± 0.20 a	2.19 ± 0.00 c	2.85 ± 0.01 d	8.68 ± 1.37 a	75.63 ± 1.20 c
	H12	218.43 ± 0.79 ab	30.32 ± 0.09 b	46.11 ± 0.57 c	0.98 ± 0.09 ab	11.24 ± 0.17 bc	2.08 ± 0.01 b	1.71 ± 0.04 a	7.59 ± 0.99 a	76.41 ± 0.67 cd
Full-heading	H21	206.26 ± 15.64 a	33.35 ± 0.13 d	42.81 ± 0.44 a	1.32 ± 0.53 b	12.07 ± 0.27 d	1.99 ± 0.01 a	1.58 ± 0.09 a	6.87 ± 0.08 a	76.16 ± 0.39 cd
	H22	194.20 ± 7.47 a	31.53 ± 0.72 c	45.99 ± 0.21 c	0.97 ± 0.17 ab	10.98 ± 0.09 b	2.09 ± 0.01 b	1.73 ± 0.10 a	6.71 ± 0.70 a	77.52 ± 0.71 d
Maturity	H31	192.27 ± 16.57 a	30.22 ± 0.24 b	44.68 ± 0.18 b	0.81 ± 0.07 ab	12.16 ± 0.18 d	2.70 ± 0.05 d	2.23 ± 0.18 b	7.22 ± 0.38 a	74.89 ± 0.06 bc
	H32	217.38 ± 13.07 ab	28.04 ± 0.73 a	45.89 ± 0.36 c	0.48 ± 0.25 a	11.55 ± 0.19 c	2.74 ± 0.09 d	2.63 ± 0.13 c	8.67 ± 0.60 a	73.93 ± 0.52 ab
Background	H40	201.11 ± 1.40 a	29.42 ± 1.03 a	44.19 ± 0.45 b	0.73 ± 0.04 ab	13.00 ± 0.76 e	2.97 ± 0.20 e	2.31 ± 0.21 b	7.40 ± 0.72 a	73.60 ± 1.46 a
Panicle differentiation	L11	146.61 ± 13.43 a	33.71 ± 0.94 a	40.11 ± 0.15 c	1.06 ± 0.09 a	11.84 ± 0.60 b	2.26 ± 0.08 ab	2.49 ± 0.12 ab	8.53 ± 0.87 a	73.82 ± 0.97 a
	L12	167.55 ± 18.64 ab	32.23 ± 0.63 a	41.79 ± 0.31 d	0.92 ± 0.24 a	10.49 ± 0.47 a	2.16 ± 0.03 a	3.31 ± 0.58 c	9.09 ± 0.69 a	74.02 ± 0.72 a
Full-heading	L21	167.69 ± 19.92 ab	37.72 ± 1.11 b	36.46 ± 0.40 a	0.86 ± 0.13 a	13.44 ± 0.43 c	2.09 ± 0.03 a	1.93 ± 0.09 a	7.50 ± 0.55 a	74.18 ± 1.06 a
	L22	156.68 ± 12.40 a	32.88 ± 1.03 a	41.23 ± 0.34 d	0.90 ± 0.54 a	10.88 ± 0.63 a	2.40 ± 0.07 ab	3.16 ± 0.47 bc	8.56 ± 0.72 a	74.11 ± 1.33 a
Maturity	L31	161.39 ± 21.96 ab	33.15 ± 1.74 a	38.21 ± 0.54 b	0.56 ± 0.04 a	12.42 ± 0.49 bc	2.51 ± 0.32 b	3.05 ± 0.27 bc	10.12 ± 3.30 a	71.36 ± 2.28 a
	L32	197.07 ± 6.59 b	33.55 ± 0.76 a	39.07 ± 0.15 c	0.82 ± 0.52 a	13.06 ± 0.47 c	2.55 ± 0.03 b	3.43 ± 0.32 c	7.50 ± 0.15 a	72.63 ± 0.61 a
Background	L40	134.17 ± 10.59 a	31.43 ± 0.86 a	39.79 ± 0.98 c	0.43 ± 0.02 a	13.48 ± 0.49 c	2.88 ± 0.16 c	3.37 ± 0.18 c	8.61 ± 0.61 a	71.22 ± 0.31 a
Sample Type (T)		8.713**	7.541*	24.090**	4.237	1.317	0.311	6.403*	1.283	19.021**
Stage (S)		0.804	4.655*	2.218	12.122**	3.568	20.516**	2.878	1.110	19.047**
T*S		5.554**	8.857**	26.644**	0.599	13.881**	5.749**	7.897**	1.994	1.041

^1^The data are expressed as mean ± SD (n = 3). Different letters within columns indicate significant differences (p < 0.05).

Asterisks indicated the factor significantly explaned the varaition among the sanples (*p < 0.05; **p < 0.01).

**Table 3:** Explanation of the factors used in RDA with significant correlationto PLFA and enzyme activities data using Monte Carlo permutational tests.

PLFA^1^			Enzyme^2^			Enzyme^3^		
H&L	H	L	H&L	H	L	H&L	H	L
TP (35%)***	TC (31%)***	pH (25%)***	TN (15%)***	AK (18%)**	AK (32%)**	GN (25%)***	Act (26%)***	Act (19%)**
MOI (14%)***	MOI (16%)***	TN (18%)***	AK (12%)**	MOI (17%)**	TK (16%)**	Fun (8%)**		
AK (4%)*	AK (11%)**	AN (10%)**	MOI (6%)*	TK (13%)**	AN (15%)**	Act (5%)*		
pH (4%)*	pH (8%)**				pH (7%)**			
					TC (5%)*			

Asterisks indicated the factor significantly explaned the varaition among the sanples (*p < 0.05; **p < 0.01; ***p < 0.001).

**Table 4:** Carbon substrates most heavily loaded on first two principal components (PC) in the PCA analysis of Biolog Eco-Plate data.

Field H		Field L	
PC1	PC2	PC1	PC2
4-Hydroxybenzoic Acid	Phenylethylamine	Phenylethylamine	4-Hydroxybenzoic Acid
Tween 80	Itaconic Acid	2-Hydroxybenzoic Acid	Tween 80
Glycogen	D-Malic Acid	γ-Hydroxybutyric Acid	L-Arginine
α-Ketobutyric Acid	L-Threonine	D-Xylose	L-Asparagine
L-Phenylalanine	D-Galacturonic Acid	i-Erythritol	L-Serine
Glycyl-L-Glutamic Acid	i-Erythritol	D-Cellobiose	D-Mannitol
D-Mannitol	α-D-Lactose	α-D-Glucose-1-Phosphate	N-Acetyl-D-Glucosamine
D-Cellobiose			
Total variation accounted by PC1 or PC2			
21.3%	14.1%	22.2%	20.9%

The loading was >0.07 or <−0.07.

**Table 5:** Diversity indexes calculated from 31 sole carbon source substrates utilization data For diversity analysis, original data at the experiment end points was normalized to AWCD.

Stage	Sample Type	Shannon (H)	Simpson (1/D)	Mclntosh (U)
Panicle differentiation	H11	3.09 ± 0.04 b	0.95 ± 0.00 b	4.68 ± 0.10 c
	H12	2.84 ± 0.09 a	0.93 ± 0.00 a	4.17 ± 0.04 b
	L11	2.86 ± 0.06 a	0.93 ± 0.00 a	4.07 ± 0.16 b
	L12	2.72 ± 0.03 a	0.94 ± 0.01 a	3.33 ± 0.12 a
Full-heading	H21	2.84 ± 0.04 b	0.93 ± 0.00 b	3.85 ± 0.16 c
	H22	2.51 ± 0.07 a	0.90 ± 0.00 a	1.65 ± 0.13 a
	L21	2.73 ± 0.06 b	0.93 ± 0.00 b	3.50 ± 0.14 c
	L22	3.07 ± 0.08 c	0.94 ± 0.00 b	2.88 ± 0.55 b
Maturity	H31	3.02 ± 0.05 c	0.94 ± 0.00 b	3.64 ± 0.17 c
	H32	2.79 ± 0.04 b	0.93 ± 0.00 b	3.10 ± 0.06 b
	L31	2.72 ± 0.12 b	0.92 ± 0.02 ab	2.85 ± 0.26 b
	L32	2.56 ± 0.07 a	0.90 ± 0.01 a	2.04 ± 0.36 a
Background	H40	2.66 ± 0.08	0.92 ± 0.00	2.02 ± 0.61
	L40	2.83 ± 0.06	0.93 ± 0.00	2.73 ± 0.19
Sample Type (T)		0.682	0.848	2.338
Stage (S)		0.436	1.029	5.537*
T*S		24.473**	10.244**	15.037**

^1^Values represent the mean (n = 3) with the standard variance (STDEV). Different letters within columns indicate significant differences (p < 0.05) at a single growth stage. Asterisks indicated the factor significantly explaned the varaition among the sanples (*p < 0.05; **p < 0.01).

**Table 6:** Soil enzyme activities. Ure, Urease; Pho, Alkali phosphatas; Ary, Aryl sulfatase; Inv, Invertase.

Stage	Sample Type	Pho (mg/g)	Inv (mg/g)	Ure (μg/g)	Ary (μg/g)
Panicle differentiation	H11	0.95 ± 0.06 a	6.27 ± 0.63 a	37.22 ± 1.47 b	50.63 ± 3.56 a
	H12	0.97 ± 0.05 a	7.40 ± 0.74 a	31.36 ± 3.69 a	55.39 ± 6.15 a
	L11	1.24 ± 0.06 b	7.01 ± 0.90 a	36.81 ± 0.12 b	57.93 ± 3.83 a
	L12	1.24 ± 0.09 b	9.79 ± 0.64 b	38.81 ± 2.15 b	56.40 ± 3.00 a
Full-heading	H21	0.97 ± 0.05 a	4.29 ± 0.26 a	40.26 ± 1.82 c	73.21 ± 11.90 a
	H22	0.93 ± 0.04 a	3.99 ± 0.33 a	21.65 ± 4.25 a	71.05 ± 1.12 a
	L21	0.88 ± 0.02 a	3.63 ± 0.27 a	32.71 ± 2.12 b	84.68 ± 9.47 a
	L22	1.12 ± 0.11 b	5.77 ± 3.50 a	43.75 ± 5.25 c	74.04 ± 17.67 a
Maturity	H31	1.05 ± 0.11 ab	7.85 ± 3.38 a	41.93 ± 6.44 b	71.26 ± 16.22 a
	H32	0.96 ± 0.06 a	7.36 ± 2.72 a	42.89 ± 4.20 b	64.08 ± 5.61 a
	L31	1.08 ± 0.07 ab	9.49 ± 2.37 a	31.66 ± 6.15 ab	75.55 ± 15.47 a
	L32	1.16 ± 0.00 b	7.47 ± 2.83 a	27.80 ± 2.48 a	80.87 ± 3.00 a
Background	H40	0.92 ± 0.03	6.04 ± 0.69	34.26 ± 2.98	93.44 ± 2.48
	L40	0.96 ± 0.08	4.09 ± 0.25	31.94 ± 1.04	108.33 ± 6.41
Sample Type (T)		3.047	0.261	0.473	8.826**
Stage (S)		2.931	4.558*	0.088	43.391**
T*S		7.052**	30.208**	26.479**	1.660

^1^Values represent the mean (n = 3) with the standard variance (STDEV). Different letters with NSK tests indicate significant differences (p < 0.05) at a single growth stage.

Asterisks indicated the factor significantly explaned the varaition among the sanples (*p < 0.05; **p < 0.01).
